# Diagnostic confirmation of mild traumatic brain injury by diffusion tensor imaging: a case report

**DOI:** 10.1186/1752-1947-6-66

**Published:** 2012-02-16

**Authors:** Ranga Krishna, Michael Grinn, Nicholas Giordano, Magesh Thirunavukkarasu, Prasanna Tadi, Shibani Das

**Affiliations:** 1 Medical Clinic of New York, 1513 Voorhies Avenue, Brooklyn, New York 11235, USA

## Abstract

**Introduction:**

Traumatic brain injury is a form of acquired brain injury that results from sudden trauma to the head. Specifically, mild traumatic brain injury is a clinical diagnosis that can have significant effects on an individual's life, yet is difficult to identify through traditional imaging techniques.

**Case presentation:**

This is the case of a 68-year-old previously healthy African American woman who was involved in a motor vehicle accident that resulted in significant head trauma. After the accident, she experienced symptoms indicative of mild traumatic brain injury and sought a neurological consultation when her symptoms did not subside. She was initially evaluated with a neurological examination, psychological evaluation, acute concussion evaluation and a third-party memory test using software from CNS Vital Signs for neurocognitive function. A diagnosis of post-concussion syndrome was suggested. Diffusion tensor imaging revealed decreased fractional anisotropy in the region immediately adjacent to both lateral ventricles, which was used to confirm the diagnosis. Fractional anisotropy is a scalar value between zero and one that describes the degree of anisotropy of a diffusion process. These results are indicative of post-traumatic gliosis and are undetectable by magnetic resonance imaging. Our patient was treated with cognitive therapy.

**Conclusion:**

Minor traumatic brain injury is a common injury with variable clinical presentation. The system of diagnosis used in this case found a significant relationship between the clinical assessment and imaging results. This would not have been possible using traditional imaging techniques and highlights the benefits of using diffusion tensor imaging in the sub-acute assessment of minor traumatic brain injury.

## Introduction

Traumatic brain injury (TBI) is a form of acquired brain injury resulting from sudden trauma to the head. In the general population, motor vehicle accidents (MVAs) are the leading cause of TBI [1]. Symptoms of TBI can range from mild to moderate to severe. A person with mild TBI (MTBI) may or may not experience a loss of consciousness. Other symptoms of MTBI include headache, confusion, lightheadedness, dizziness, blurry vision, tinnitus, dysgeusia, fatigue, changes in sleep patterns or behavior and impairment of memory or cognition. Despite such variable presentations, patients with MTBI are assessed clinically. This is due to the inability of a magnetic resonance imaging (MRI) to detect positive findings in a majority of patients. A novel imaging software, diffusion tensor imaging (DTI), is able to display neural tract deficits that are undetectable by MRI. It does so by evaluating the fractional anisotropy (FA) of neurons. Changes in FA reflect changes in the principal direction of the diffusion tensor and can be used to evaluate the condition of the white-matter connectivity of the brain [2,3]. A decreased FA is associated with MTBI [4]. In this approach, we examined our patient's symptoms on various levels to fully understand the utility of DTI in patients with MTBI. A table of the working definitions in this study is found in Table 1. Since an accurate diagnosis is key in determining the management and predicting the outcome of patients with head trauma, it is important to use all modalities available for diagnosis, specifically DTI.

**Table 1:** Definition of terms.

Term	Definition
**Traumatic brain injury**	A form of acquired brain injury resulting from sudden trauma to the head. Complications of such an injury can result in permanent or temporary deficits in cognitive, physical and psychosocial functions.
**Magnetic resonance imaging (MRI)**	Currently the most sensitive imaging test of the head commonly used in clinical practice. An MRI uses a magnetic field and radiofrequency pulses to provide detailed images of soft tissues and internal body structures.
**Diffusion tensor imaging (DTI)**	A new technology capable of providing images of neural tracts. DTI accomplishes this by measuring the restricted diffusion of water through tissues.
**Fractional anisotropy (FA)**	A scalar value between zero and one that describes the degree of anisotropy of a diffusion process. A value of zero means that diffusion is isotropic, that is, it is unrestricted (or equally restricted) in all directions. A value of one means that diffusion occurs only along one axis and is fully restricted in all other directions
**Neuropsychological evaluation**	An evaluation in which a trained neuropsychologist can acquire data about a subject's cognitive, motor, behavioral, language and executive functioning. Such information can lead to a diagnosis as well as to the localization of abnormalities in the central nervous system.

## Case presentation

This is the case of a previously healthy African American woman who was involved in an MVA at the age of 68 years. She was a Certified Nursing Assistant at the time and had no prior history suggestive of neurodegenerative disease. Our patient was in the backseat when her vehicle struck another. On impact she was thrown forward, hitting her head (right frontal-temporal region), knee and hands. She reported feeling lightheaded immediately after impact.

Upon arrival at our emergency department, our patient was conscious with a Glasgow Coma Scale of 15. However, it was soon noted that she was experiencing anterograde amnesia. Our patient was thus administered a cognitive questionnaire. She was fully oriented to person, place and time. Her remote memory appeared to be intact, and on the immediate recall exam she scored three out of four. As for category fluency, she was able to name 12 animals in 30 seconds. Overall, our patient scored 40 out of 50, indicating mild cognitive impairment.

An acute concussion evaluation, which is a standardized evaluation presented by the Center for Disease Control for quick and reliable identification of TBI, was then administered [5]. In our emergency department, she scored 11 out of 22. Physical exertion was noted to exacerbate her symptoms and she was reported to be acting significantly different from her usual self, scoring five on a zero-to-six scale. Our patient was thus diagnosed (International Classification of Diseases, ICD-10) with concussion with no loss of consciousness (code 850.0). She was admitted for two days following the accident due to her injuries.

Our patient was administered a neurological examination one month later when her symptoms failed to subside. At the initial visit she presented with headache and dizziness. Additionally, our patient had several symptoms indicative of cervical and lumbar disk syndrome, unrelated to MTBI. These symptoms included upper back pain, neck pain and lower back pain. Her general pain scale was rated eight out of ten. Examinations of her cranial nerves and brainstem reflexes were unremarkable.

Based on a memory test provided by CNS Vital Signs, which is a neurocognitive third-party software, it was noted that our patient's memory had been affected by the MVA [6]. The memory test involves a combination of seven standard tests, a verbal memory test, visual memory test, finger tapping test, symbol digit coding, Stroop test, shifting attention test and continuous performance test. Our patient was profiled in the very low range of all domain scores, except for reaction time in which she performed in the average range.

Our patient then underwent a current procedure terminology code 90801: Psychiatric Diagnostic Interview Examination three months after the MVA. During the examination, our patient demonstrated excessive anxiety regarding the MVA. She demonstrated good abstract reasoning ability and a good general fund of information. Her affect was appropriate and her speech was goal-directed. However, her distress about her cognitive problems and physical pain indicated a need for psychotherapy. Our patient's diagnosis according to the Diagnostic and Statistics Manual of Mental Disorders-IV was 309.28: adjustment disorder with mixed anxiety and depressed mood. She also reported sleep disturbance and sporadic eating habits, as well as generalized tension. Her most compelling complaint was the onset of problems with her memory, concentration, reading comprehension and verbal comprehension. Such symptoms are suggestive of ICD-9 310.20: post-concussion syndrome. Her Global Assessment of Functioning score was 45, indicating serious impairment.

**Figure 1:**
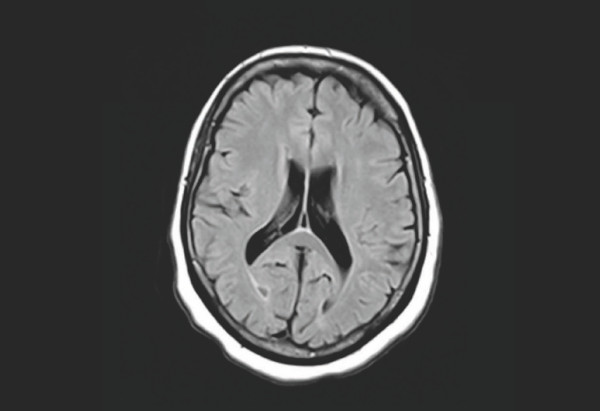
**Three-dimensional diffusion tensor image of our patient**. These images demonstrate a decreased Fractional anisotropy in the periventricular white matter adjacent to both lateral ventricles, indicating post-traumatic gliosis affecting the surrounding colossal fibers. fractional anisotropy reflects the degree of fiber organization, fiber directional coherence or fiber integrity. Areas with decreased fractional anisotropy are displayed in red.

A multiplanar and multisequential MRI examination of her brain was performed to examine the clinical indications of headaches and her post-MVA status. A Siemens 1.5 Espree system was used with software version Syngo BR17. There was no mass effect or midline shift. There was no evidence of hemorrhage or subdural hematoma. Her craniovertebral junction was normal. MRI did not show any evidence of prior neurodegenerative disease (Figure 1).

**Figure 2:**
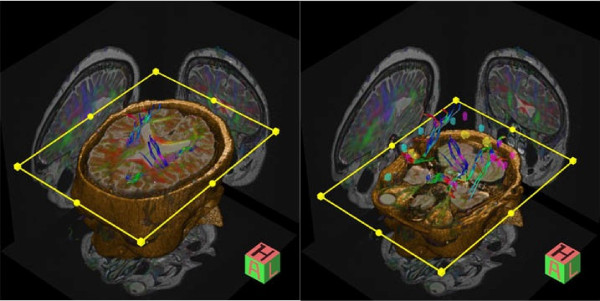
**Magnetic resonance imaging of our patient**. The transverse image is unremarkable and does not indicate minor traumatic brain injury. This is the same plane where diffusion tension imaging indicates trauma.

Tractographic diffusion tensor images were then obtained following an unremarkable MRI to search for axonal injury using the same Siemens 1.5 Espree system with the associated DTI software. Sagittal, coronal and axial sequence images of her brain were obtained. A decreased FA was discovered in the periventricular white matter (PWM) immediately adjacent to both lateral ventricles (Figure 2). These results are indicative of post-traumatic gliosis. This region reflects changes in the principal direction of the diffusion tensor and can be used to evaluate the condition of white-matter connectivity of the brain. A decreased FA in the brain is associated with MTBI [4]. The region of FA was not of interest prior to this current examination, and was discovered through the DTI study. Normal FA values have been identified by the board-certified radiologist and were compared to our patients' results to identify abnormalities.

## Discussion

TBI is a significant public health problem. An estimated 1.7 million people sustain TBIs in the United States each year [7]. TBI is frequently referred to as the silent epidemic because its complications, such as impairment of cognition, may not at first be apparent. In addition, awareness of TBI among the general public is limited. TBI is commonly seen in children, adolescents and adults aged 65 years and older. A TBI may be caused by a bump, blow or jolt to the head that causes disruption to the normal function of the brain [8,9]. Falls, sporting injuries and MVAs are the most likely causes of TBI. MTBI accounts for 75% of all TBIs in the United States annually [8].

As the Center for Disease Control states, there are many clinical indications that can lead to the diagnosis of MTBI. However, there has been minimal discussion regarding the correlation of these clinical indications with medical imaging results [7]. As opposed to moderate or severe TBI, MTBI is difficult to diagnose since MRI and positron emission tomography (PET) scans are unremarkable in a majority of MTBI cases. MRI and PET scans are incapable of demonstrating pathology unrelated to structural abnormalities; research shows that 43% to 68% of MTBI patients have normal structural scans on MRI [10]. Although this may be due to there being no structural brain damage, it should also be noted that this technology might not be sufficient. Due to this, the diagnosis of MTBI remains, up until this point, a clinical one, where patients' complaints are not backed up by physiological evidence.

Our patient was involved in an MVA which resulted in significant head trauma. After the accident, our patient complained of symptoms such as post-traumatic headache, dizziness, cognitive deficits, anterograde amnesia and sleep disturbances. Such symptoms, along with a history of head trauma, are indicative of MTBI [11]. To assess this patient acutely, a neurological examination, a Cognitive Questionnaire, and an Acute Concussion Evaluation were given in our emergency department. One month later, when symptoms failed to subside, a neurological examination, psychological examination, and CNS Vital Signs Memory Test were used to assess our patient. She was found to have a significant memory deficit, post-concussion syndrome and adjustment disorder with mixed anxiety and depressed mood. An MRI was then performed which failed to detect any abnormalities. It wasn't until a DTI study was performed that an imaging study yielded a positive finding. The study could have benefited from follow-up examination, which was not available. This highlights the importance of retrieving follow-up clinical data in order to better understand the full capability of DTI in the treatment of a patient with MTBI.

DTI revealed a decreased FA in the white matter immediately adjacent to both lateral ventricles, which indicates trauma to the PWM surrounding the lateral ventricles. These results are indicative of post-traumatic gliosis affecting the surrounding colossal fibers [4,12] (Figure 1). This finding offers an explanation for our patient's clinical picture, as PWM lesions are associated with reduced cognitive function [4]. This exact area appeared unremarkable on MRI (Figure 2). This is a breakthrough, in the respect that physicians may now have the ability to assess MTBIs using an imaging technique capable of showing objective findings that can account for a patient's clinical presentation.

## Conclusion

MTBI is a significant issue in the United States, as it results in chronic effects in 1.7 million people each year. The direct and indirect costs of TBI in the US have been estimated to be upward of $60 billion annually [1]. Therefore, a cost efficient, systematic method of diagnosing MTBI could serve the individual and the medical community through increasing specificity of care, leading to a reduction in the amount of time that an individual is not able to work. This increase in treatment specificity and increased quality of care as a secondary prevention strategy is important from a public health perspective. In this case, DTI served as a powerful diagnostic tool, providing imaging results that offered an explanation for our patient's clinical picture. To explore these findings, a larger study should be conducted to better understand the potential role of DTI in the diagnosis and assessment of MTBI.

## Consent

Written informed consent was obtained from the patient for publication of this case report and any accompanying images. A copy of the written consent is available for review by the Editor-in-Chief of this journal.

## Competing interests

The authors declare that they have no competing interests.

## Authors' contributions

RK provided and interpreted the neurological consultation, as well as being a primary editor for the case report. MT was the lead in data accrual, providing the memory test and acute concussion evaluation, along with interpretations, and was an editor of the manuscript. PT was involved in data analysis, and a major contributor to the overall manuscript editing. SD was responsible for the neuropsychological data accrual and write-up. NG and MG were major editors and writers, provided a literary review for references and were a part of the data analysis team. All authors read and approved the final manuscript.
